# Reply to: 'Postgraduate family medicine training in Southeast Asia' by Wattanapisit et al. (2025)

**DOI:** 10.51866/lte.979

**Published:** 2025-09-02

**Authors:** Schawanya Kaewpitoon Rattanapitoon, Chutharat Thanchonnang, Nathkapach Kaewpitoon Rattanapitoon

**Affiliations:** 1 MD, FCFP, FMC Medical Center, Nakhon Ratchasima, Thailand. Email: schawanya.ratt@g.sut.ac.th; 2 BSc, PhD candidate, FMC Medical Center, Nakhon Ratchasima, Thailand.; 3 BSc (Public Health), BSc, (Occupational Health), MSc, PhD FMC Medical Center, Nakhon Ratchasima, Thailand.

**Keywords:** Family medicine training, Southeast Asia, Regional harmonisation, Digital learning, Undergraduate medical education, Health workforce development

**Dear editor,**

We commend Wattanapisit et al.^1^ for their comprehensive overview of postgraduate family medicine training across eight Southeast Asian countries. Their work represents a crucial step towards documenting regional diversity in training structures, policy commitment and institutional evolution. It also offers a foundational resource for comparative learning across contexts.

While the article provides valuable country-level insights, we wish to extend the discussion by highlighting three additional dimensions that may guide strategic advancement in regional family medicine development.

1. **Advancing regional harmonisation**
  Despite the varied national contexts, there is untapped potential for regional harmonisation of family medicine training. Drawing from the Bologna Process in Europe, Southeast Asia could explore frameworks for shared core competencies, mutual recognition of postgraduate qualifications and faculty exchange programmes.^2,3^ These steps would not only promote academic mobility but also address health workforce disparities and strengthen regional solidarity in primary care.
2. **Leveraging digital innovation to bridge the rural gap**
  While Cambodia’s online diploma initiative is mentioned, the transformative potential of digital learning across the region remains underexplored. Given the limited number of family medicine trainers and urban-centric training facilities, a regionally coordinated digital learning platform - potentially under ASEAN or WONCA Asia Pacific - could expand access to high- quality training, particularly in underserved rural areas.^4,5^ Blended and asynchronous e-learning models have proven effective in low-resource settings globally and may offer scalable solutions for Southeast Asia.
3. **Cultivating early interest through undergraduate exposure**
  Workforce development in family medicine must begin upstream. Countries such as Thailand and the Philippines have embedded community-based learning and primary care principles into undergraduate medical education, positively influencing students’ specialty choices.^6^ A regional strategy that promotes early exposure to family medicine could foster stronger interest, continuity and preparedness among future trainees.

In conclusion, Wattanapisit et al.’s article provides essential groundwork for regional reflection. We propose that the next phase of progress lies in integration - through shared educational standards, digital innovation and early-stage engagement in training pathways. These strategies can collectively elevate the role of family medicine in building resilient, equitable health systems across Southeast Asia.
